# Use of RDTs to improve malaria diagnosis and fever case management at primary health care facilities in Uganda

**DOI:** 10.1186/1475-2875-9-S2-P18

**Published:** 2010-10-20

**Authors:** Daniel J Kyabayinze, Caroline Asiimwe, Damalie Nakanjako, Jane Nabakooza, Helen Counihan, James K Tibenderana

**Affiliations:** 1 Malaria Consortium, P.O.Box 25308 Kampala

## Background

Early and accurate diagnosis of malaria followed by prompt treatment reduces the risk of severe disease in malaria endemic regions. Presumptive treatment of malaria is widely practised where microscopy or rapid diagnostic tests (RDTs) are not readily available. With the introduction of artemisinin-based combination therapy (ACT) for treatment of malaria in many low-resource settings, there is need to target treatment to patients with parasitologically confirmed malaria in order to improve quality of care, reduce over consumption of anti-malarials, reduce drug pressure and in turn delay development and spread of drug resistance. This study evaluated the effect of malaria RDTs on health workers' anti-malarial drug (AMD) prescriptions among outpatients at low level health care facilities (LLHCF) within different malaria epidemiological settings in Uganda.

## Methods

All health workers (HWs) in 21 selected intervention (where RDTs were deployed) LLHF were invited for training on the use RDTs. All HWs were trained to use RDTs for parasitological diagnosis of all suspected malaria cases irrespective of age. Five LLHCFs with clinical diagnosis (CD only) were included for comparison. Subsequently AMD prescriptions were compared using both a 'pre - post' and 'intervention - control' analysis designs. In-depth interviews of the HWs were conducted to explore any factors that influence AMD prescription practices.

## Results

A total of 166,131 out-patient attendances (OPD) were evaluated at 21 intervention LLHCFs. Overall use of RDTs resulted in a 38% point reduction in AMD prescriptions. There was a two-fold reduction (RR 0.62, 95% CI 0.55-0.70) in AMD prescription with the greatest reduction in the hypo-endemic setting (RR 0.46 95% CI 0.51-0.53) but no significant change in the urban setting (RR1.01, p-value = 0.820). Over 90% of all eligible OPD patients were offered a test. An average of 30% (range 25%-35%) of the RDT-negative fever patients received AMD prescriptions. When the test result was negative, children under five years of age were two to three times more likely (OR 2.6 p-value <0.001) to receive anti-malarial prescriptions relative to older age group. Of the 63 HWs interviewed 92% believed that a positive RDT result confirmed malaria, while only 49% believed that a negative RDT result excluded malaria infection.

## Conclusion

Use of RDTs resulted in a 2-fold reduction in anti-malarial drug prescription at LLHCFs. The study demonstrated that RDT use is feasible at LLHCFs, and can lead to better targeting of malaria treatment. Nationwide deployment of RDTs in a systematic manner should be prioritised in order to improve fever case management. The process should include plans to educate HWs about the utility of RDTs in order to maximize acceptance and uptake of the diagnostic tools and thereby leading to the benefits of parasitological diagnosis of malaria.

